# Functional Supramolecular Gels Based on the Hierarchical Assembly of Porphyrins and Phthalocyanines

**DOI:** 10.3389/fchem.2019.00336

**Published:** 2019-05-15

**Authors:** Xuenan Feng, Chenxi Liu, Xiqian Wang, Yuying Jiang, Gengxiang Yang, Rong Wang, Kaishun Zheng, Weixiao Zhang, Tianyu Wang, Jianzhuang Jiang

**Affiliations:** Beijing Key Laboratory for Science and Application of Functional Molecular and Crystalline Materials, Department of Chemistry, University of Science and Technology Beijing, Beijing, China

**Keywords:** supramolecular assembly, supramolecular gels, porphyrins, phthalocyanine, π-conjugated systems, functional soft matters

## Abstract

Supramolecular gels containing porphyrins and phthalocyanines motifs are attracting increased interests in a wide range of research areas. Based on the supramolecular gels systems, porphyrin or phthalocyanines can form assemblies with plentiful nanostructures, dynamic, and stimuli-responsive properties. And these π-conjugated molecular building blocks also afford supramolecular gels with many new features, depending on their photochemical and electrochemical characteristics. As one of the most characteristic models, the supramolecular chirality of these soft matters was investigated. Notably, the application of supramolecular gels containing porphyrins and phthalocyanines has been developed in the field of catalysis, molecular sensing, biological imaging, drug delivery and photodynamic therapy. And some photoelectric devices were also fabricated depending on the gelation of porphyrins or phthalocyanines. This paper presents an overview of the progress achieved in this issue along with some perspectives for further advances.

## Introduction

**Figure 1:**
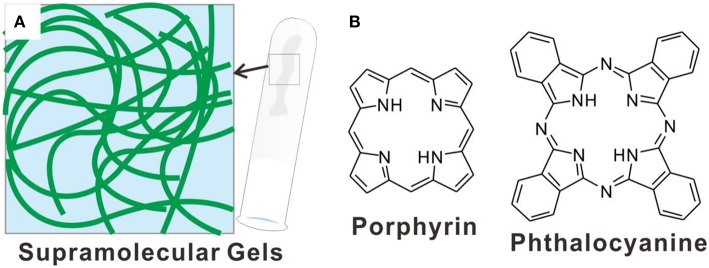
**(A)** Schematic illustration showing nanostructures of typical supramolecular gels; **(B)** molecular structures of porphyrin and phthalocyanine.

Supramolecular gels (Steed, 2010; Buerkle and Rowan, 2012; Smith, 2012; Yu et al., 2013), in which organic molecules self-assemble into nano/microstructures and immobilize the solvents (Figure 1A), are attracting increased interests in different fields (Edwards and Smith, 2014; Shen et al., 2015; Kaufmann et al., 2016). Depending on the solvent included into the gels systems, the supramolecular gels can be either hydrogels or organogels. As the soft matters depending on non-covalent interactions, supramolecular gels not only show very nice dynamic, reversible and stimuli-responsive nature (Foster et al., 2010; Zhao et al., 2013; Wang et al., 2015a, 2018), but also have versatile applications in the field of life and materials sciences (Bhattacharya and Samanta, 2016; Goujon et al., 2017; Yang et al., 2017; Das et al., 2018). Based on the self-assembly of various molecular systems, supramolecular gels with delicate structures have been designed and developed (Yu et al., 2014; Silverman et al., 2017; Mallia and Weiss, 2018). One of the most important advantages of supramolecular gels can be their capability of incorporating different functional motifs via varied, sometimes very simple processes (Buerkle and Rowan, 2012). For example, the fabrication of supramolecular gels containing π-conjugated systems can be either from the synthesis and subsequent self-assembly of π-conjugated gelators or the co-assembly within carefully designed multicomponent gelating systems, wherein π-conjugated functional groups can be included by using simple molecular building blocks (Bhattacharya and Samanta, 2016).

Tetrapyrrole macrocycle based π-conjugated systems, such as porphyrin and phthalocyanine derivatives (Figure 1B), are very famous rigid and aromatic molecular building blocks with special electronic structures (Buchler and Ng, 2000; Mack and Stillman, 2003). Porphyrin is one of the most important natural pigments, which sets up the basis of hemoglobin and chloroplasts, realizes many significant functions, from carrying oxygen to photosynthesis (Drain et al., 2009; Severance and Hamza, 2009; Kundu and Patra, 2017). Both porphyrin and phthalocyanine derivatives have distinctive photophysical, photochemical and electrochemical properties (Bian et al., 2011; Lauceri et al., 2011; Hasobe, 2014). And the application of these tetrapyrrole macrocycle based π-conjugated systems have been developed in the field of catalysis (Feiters et al., 2000; Zhang et al., 2017), molecular sensing (Paolesse et al., 2017), organic semiconductor (Jiang et al., 2018), solar cells (Urbani et al., 2014), and photodynamic therapy (Liu et al., 2013; Giuntini et al., 2014; Moylan et al., 2015; Chen et al., 2017; Li et al., 2018a; Zhu et al., 2018). It is conceivable that these different functions should be dependent on both the molecular structures and supramolecular architectures of porphyrin and phthalocyanine derivatives. Especially, the systems derived from the hierarchical assembly of porphyrins and phthalocyanines are becoming more and more important. For the achievement of so many applications, the cooperative organization of molecular building blocks plays very important role (Guo et al., 2014; Zhao et al., 2014; Zhang et al., 2015; Geng et al., 2017, 2018; Yang et al., 2018c; Chang et al., 2019; Li et al., 2019b). And supramolecular gels with plentiful nanostructures and properties can be good carriers for the fabrication of porphyrin and phthalocyanine assemblies. Moreover, based on supramolecular gels, the porphyrin and phthalocyanine assemblies with stimuli-responsive and dynamic properties can also be expected. Most importantly, the good biocompatible nature of supramolecular gels has been demonstrated, which guarantee the biomedical applications of porphyrin and phthalocyanine assemblies.

Although the advantages of supramolecular gels based on the assembly of porphyrins and phthalocyanines are distinct, building these systems has been demonstrated to be not easy. This situation is partly due to the difficulty of the synthesis of gelators containing porphyrin or phthalocyanine motifs (Ishi-i and Shinkai, 2005). Especially, the modification of phthalocyanine rings with typical functional groups for gelation, for example cholesterol, sugar and amino acids (Weiss and Terech, 2006; Weiss, 2018), is difficult. And the synthesis of porphyrin derivatives may also be confronted with some serious barriers, such as low yield, limitations on preparative scale, and difficulties in purification. On the other hand, even though introducing porphyrin or phthalocyanine motifs into supramolecular gels can be totally dependent on the hierarchical self-assembly, the network of complex non-covalent interactions and possible phase separation (Tanaka et al., 2012; Helmich and Meijer, 2013) within multicomponent supramolecular gels still should be considered. This strategy also requires carefully molecular designing to form delicate supramolecular assemblies.

In this manuscript, we will focus on the works about the construction and functions of supramolecular gels containing porphyrin and phthalocyanine derivatives. And the functions and typical applications of these systems depict a very good prospect for the further development. The supramolecular gels containing π-conjugated systems have many common characters like that of general low-molecular-weight gels. Certainly, porphyrin or phthalocyanine derivatives also can afford supramolecular gels with many new features, which expand the application of these soft matters. The gelation usually needs chiral component to be included (Weiss and Terech, 2006). However, the supramolecular gels containing only achiral molecules were also developed (Shen et al., 2015). Therefore, the supramolecular chirality of these gels systems was addressed in the paper. The mechanical properties of supramolecular gels can be changed upon the variation of gelator substituents, which were investigated in the gels systems containing porphyrin and phthalocyanines. In addition to supramolecular gels containing porphyrin and phthalocyanine derivatives, some interesting works on the polymer gels (Figure 3G) related with porphyrin or phthalocyanine assemblies were also included in this review. Comparing with supramolecular gels, polymer gels can be much more stable but may lack of some dynamic features. However, the polymer gels have been widely applicated in many fields, which also plays a leading role for the further development of supramolecular gels. Nonetheless, polymer gels containing porphyrin and phthalocyanine molecules also show some distinctive features.

## Constructing Supramolecular Gels Containing Porphyrin or Phthalocyanine Molecules

In principle, there are two strategies for introducing porphyrin and phthalocyanine derivatives into supramolecular gels. The fundamental method can be the synthesis of gelators containing porphyrin and phthalocyanine motifs. In this case, the self-assembly of these π-conjugated systems can form supramolecular gels. Another process for constructing porphyrin/phthalocyanine based supramolecular gels is not dependent on the porphyrin and phthalocyanine based gelators. Thus, even though porphyrin and phthalocyanine molecules themselves cannot form supramolecular gels, these π-conjugated molecules still can be included into the supramolecular gels via the hierarchical co-assembly of different components or just by simple mixing. The realization of this approach depends on the unique characteristics of supramolecular gels.

### Porphyrin or Phthalocyanine Gelators

The systematic research on the synthesis of porphyrin-based gelators is from Shinkai group. They have developed several types of gelators by introducing cholesterol, chiral urea groups or sugar substituents onto the phenyl groups of tetraphenylporphyrins (TPP) derivatives.

**Figure 2:**
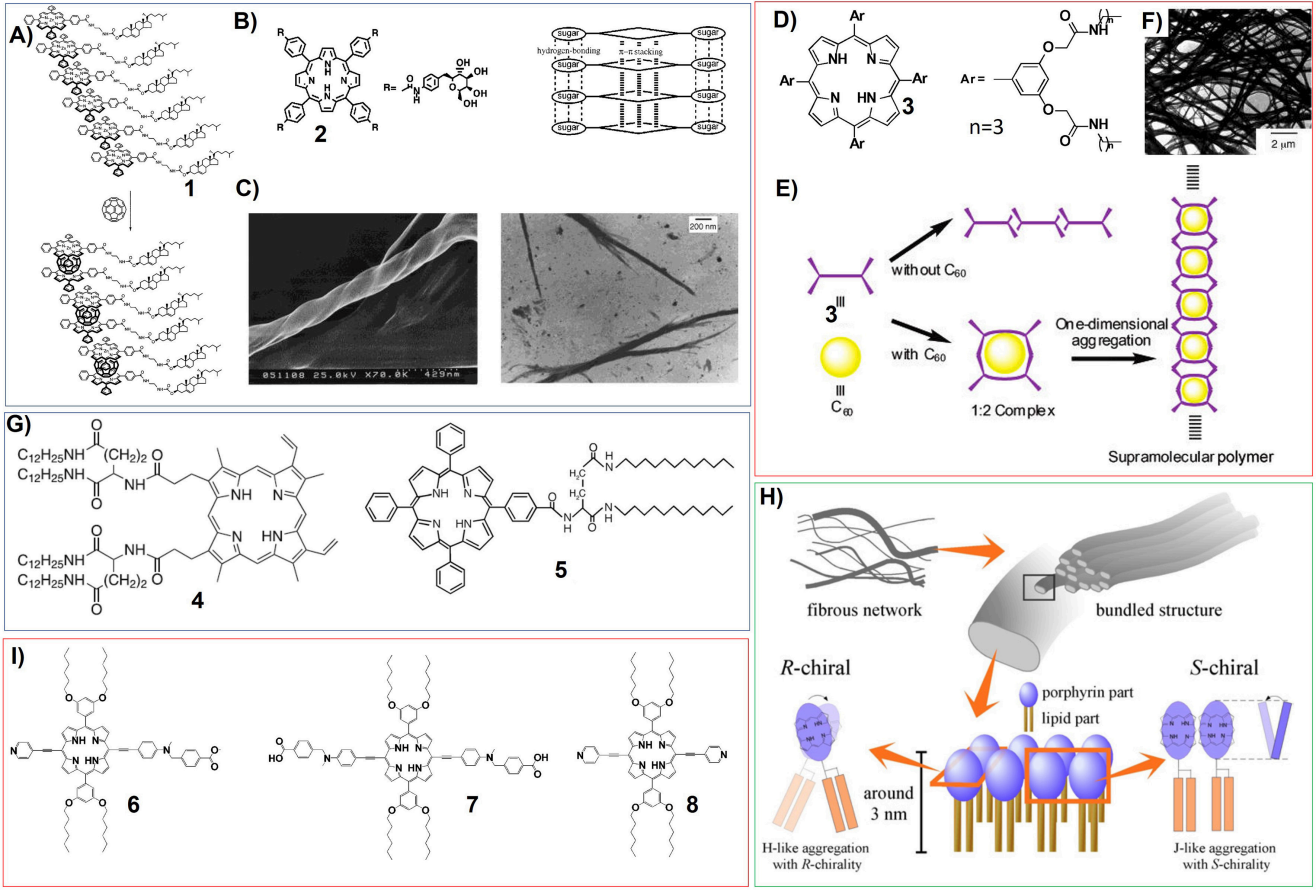
**(A)** Porphyrin appended cholesterol derivatives (1) can interact with fullerene C_60_ to form a 2:1 Zn(II) porphyrin/C_60_ sandwich complex; Reprinted with permission from Ishi-i et al. (2001). Copyright 2001 American Chemical Society. **(B)** Molecular structures of porphyrin bearing four β-D-galactopyranoside groups (2), and schematic illustration showing the molecular packing mode; Reprinted with permission from Tamaru et al. (2001). Copyright 2001 American Chemical Society. **(C)** SEM (left) and TEM (right) pictures of the xerogel of porphyrin 2; Reprinted with permission from Tamaru et al. (2001). Copyright 2001 American Chemical Society. **(D)** Molecular structures of porphyrin bearing eight amide moieties (3); Reprinted with permission from Shirakawa et al. (2003a). Copyright 2003 American Chemical Society. **(E)** Schematic illustration showing the self-assembly of porphyrin 3 as well as the co-assembly of porphyrin 3 with C_60_; Reprinted with permission from Shirakawa et al. (2003a). Copyright 2003 American Chemical Society. **(F)** TEM image of porphyrin 3 co-assembly with 0.50 equiv of C_60_; Reprinted with permission from Shirakawa et al. (2003a). Copyright 2003 American Chemical Society. **(G)** Molecular structures of porphyrin bearing didodecyl L-glutamic acid; **(H)** Schematic proposal on the ordered structure in the 5 aggregates; Reprinted with permission from Jintoku et al. (2008). Copyright 2008 Elsevier Ltd. **(I)** Molecular structures of porphyrin containing carboxylic acid and pyridine substituents.

For example, they have synthesized zinc porphyrin appended cholesterol derivatives (**1**) (Figure 2A), and found that the gelation of these porphyrins are dependent on the odd or even carbon numbers in the spacer (CH_2_)_n_, which connects the porphyrin moiety with the cholesterol groups. After adding fullerene C_60_ into the systems, the intermolecular Zn(II) porphyrin-fullerene interaction can enhance the gelation abilities of the corresponding porphyrin gelators (Figure 2A) (Ishi-i et al., 2001). Many chiral substituents associated with natural molecules have been introduced onto the porphyrin rings to synthesize porphyrin-based gelators. For instance, some brucine-appended porphyrins have been demonstrated to be nice gelators (Setnicka et al., 2002). Porphyrin gelators based on sugar substituents, which could be amphiphilic porphyrin bearing four β-D-galactopyranoside groups at its periphery (**2**) (Figure 2B), have been synthesized and studied (Tamaru et al., 2001). Compound **2** assembles into very stable organogels in DMF/alcohol mixture. The SEM and TEM measurements on the corresponding gels suggest that the self-assembly of this sugar-based porphyrin could form helical nanofibers (Figure 2C), wherein the π-π interactions between porphyrin rings and the hydrogen-bonding among sugar moieties could operate cooperatively.

Notably, for the study of porphyrin-based gelators, the co-assembly with fullerene C_60_ has attracted a lot of attention. Shinkai et al. have synthesized porphyrin bearing eight amide moieties (**3**) (Figure 2D). The cooperative non-covalent interactions including π-π interaction between porphyrin rings and the hydrogen-bonding between amide groups result in the gelation. While adding fullerene C_60_ into the systems could create one-dimensional multicapsular structures, which can be supramolecular polymer showing fibrous structures (Figures 2E,F). Both porphyrin-C_60_ interaction and hydrogen-bonding play very important role (Shirakawa et al., 2003a).

The hydrogen-bonding interactions always plays very important role for the gelation of different porphyrin-based gelators. By changing the position of amide groups around porphyrin rings, the aggregation of porphyrin building blocks can be changed from J-aggregation into H-aggregation (Shirakawa et al., 2003b). Depending on the strong hydrogen-bonding interactions from several amide groups close to each other, the porphyrins without distinct hydrophilic substituents can also form organogels. Ihara and his co-workers have synthesized porphyrins (**4**, **5**) substituted by didodecyl L-glutamic acid. These porphyrin molecules can be either derived from protoporphyrin IX (**4**) or TPP based porphyrin (**5**) (Figure 2G). These L-glutamide-functionalized porphyrin derivatives self-assemble into organogels with fibrous nanostructures. Interestingly, the packing modes of porphyrin molecules can be modulated from H-aggregation to J-aggregation with the variation of handedness of supramolecular chirality upon changing the solvent, concentration and temperature (Figure 2H) (Sagawa et al., 2002; Jintoku et al., 2008).

The asymmetric porphyrin containing both carboxylic acid and pyridine substituents (**6**) shows much better gelation properties in different organic solvents, compared with that of symmetrical reference porphyrins bearing two pyridyl substituents (**8**) or two carboxylic acid groups (**7**) (Figure 2I). In cyclohexane, the π-π interactions between porphyrin rings and the carboxylic acid-pyridine hydrogen bonding work cooperatively and render porphyrin **6** self-assemble into two-dimensional sheet-like structures (Tanaka et al., 2005).

**Figure 3:**
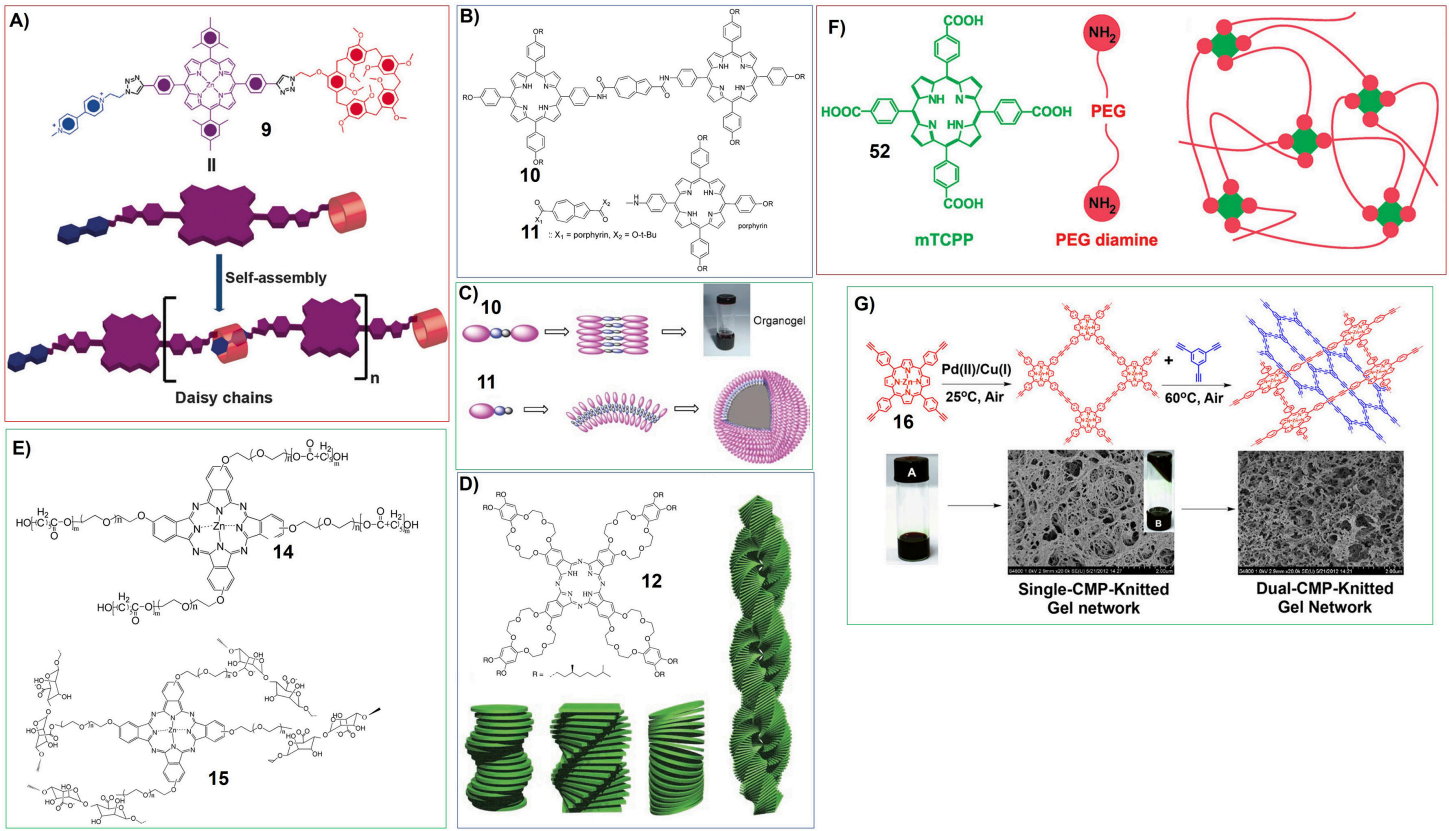
**(A)** Molecular structure and proposed self-assembly of porphyrin 9 to form linear supramolecular polymers; Reprinted with permission from Fathalla et al. (2015). Copyright 2015 Royal Society of Chemistry. **(B)** Molecular structures of porphyrin-azulene-porphyrin conjugate (10) and porphyrin-azulene conjugate (11); **(C)** Proposed mode for the formation of nanowire from 10 and vesicles from 11; Reprinted with permission from Xiao et al. (2009). Copyright 2009 Royal Society of Chemistry. **(D)** Molecular structures of phthalocyanine gelator containing crown ethers substituents and eight hydrophobic chiral tails (12) and schematic illustration showing helical aggregation formed by 12, the coiled-coil interactions introduce right-handed helical fibers form large aggregates with left-handed helicity; Reprinted with permission from Engelkamp et al. (1999). Copyright 1999 American Association for the Advancement of Science. **(E)** Molecular structures of zinc phthalocyanine polymeric gelator (14, 15); **(F)** Schematic illustration showing the reaction between PEG diamine and meso-tetrakis(4-carboxyphenyl) porphine (mTCPP) form polymer hydrogels; Reprinted with permission from Lovell et al. (2011). Copyright 2011 American Chemical Society. **(G)** Porphyrin based mesoporous polymers can uptake organic solvents to form organogels (Wu et al., 2014). Reprinted with permission from Wu et al. (2014). Copyright 2014 American Chemical Society.

Moreover, the porphyrin containing both host and guest motifs can form oganogels based on host-guest interactions. Stoddart and his co-workers have synthesized porphyrin derivative with pillar[5]arene and viologen at its 5- and 15-meso positions (**9**). The host-guest interactions between pillar[5]arenes and viologen could render the porphyrin to assemble into linear supramolecular polymer with head-to-tail manner (Figure 3A), which further form organogels at relatively higher concentrations (Fathalla et al., 2015).

Some porphyrin dimmers were also developed as supramolecular gelators. For example, the porphyrin-azulene-porphyrin conjugate (**10**) can form organogels in different organic solvents, while the corresponding monomer (**11**) self-assemble into vesicles in chloroform/methanol mixture (Figure 3B). For the self-assembly of porphyrin dimmer and monomer, the intermolecular dipole–dipole interaction of the azulene units plays very important role (Figure 3C) (Xiao et al., 2009).

Different from porphyrin-based molecular building blocks, phthalocyanine molecules usually have better planarity, which introduces stronger π-π interactions and serious aggregation (Chen et al., 2009; Gao et al., 2009). And the modification of the periphery of phthalocyanine rings is more difficult, compared with that of porphyrin derivatives. Nevertheless, some intelligent molecular design was also performed on the phthalocyanine gelators, which show some interesting characteristics.

Nolte and his co-workers have synthesized phthalocyanine containing crown ethers substituents and eight hydrophobic chiral tails (**12**) (Figure 3D). This phthalocyanine can form gels in chloroform upon self-assembly into helical fibers. The further investigations show that the molecular packing mode of phthalocyanine **12** containing (S)-chiral centers could follow a typical hierarchical process. Firstly, the stacking of phthalocyanine 12 along clockwise orientation forms the fibers with right-handed helicity. And then, coiled-coil interactions between different right-handed helical fibers could form large aggregates with left-handed helicity (Figure 3D) (Engelkamp et al., 1999).

Due to the strong π-π interactions between phthalocyanine molecules, some very simple phthalocyanine molecules were also found to form organogels. For example, tetra-n-butyl peripheral substituted copper(II) phthalocyanine (**13**) can form organogels in 1,2-dichlorobenzene upon ultrasonication (Figure 14C) (Xu et al., 2016).

For the fabrication of phthalocyanine-based soft matters, polymer gels have been employed. And the polymers containing phthalocyanine motifs have been developed. For example, the reaction between zinc phthalocyanine conjugated poly(ethylene glycol) (PEG) and ε-caprolactone produces a type of copolymer (**14**), which could form hydrogels (Dong et al., 2016a). And the zinc phthalocyanine conjugated poly(ethylene glycol) (PEG) with hydroxyl groups at the end can also be connected with alginate (**15**). Copolymer **15** can form hydrogels, which show near infrared fluorescence (Liang et al., 2017a; Figure 3E). Actually, poly(ethylene glycol) (PEG) substituents were often utilized for building porphyrins and phthalocyanines polymeric hydrogels. For instance, the reaction between PEG diamine and meso-tetrakis(4-carboxyphenyl) porphine (mTCPP) could form stable polymer hydrogels (Lovell et al., 2011; Figure 3F).

### Multicomponent Supramolecular Gels Containing Porphyrin or Phthalocyanine Molecules

The supramolecular gels containing porphyrin or phthalocyanine building blocks were also fabricated by using multicomponent systems (Cornwell et al., 2015; Draper et al., 2015; Kar and Ghosh, 2015; Versluis et al., 2016). In general, these multicomponent supramolecular gels can be divided into two types. One is the special supramolecular gels in which the gelators can be some co-assemblies including porphyrin/phthalocyanine molecular building blocks. While another type of supramolecular gels is not dependent on the assembly of porphyrin/phthalocyanine molecules. These π-conjugated molecules can be mixed into supramolecular gels formed by other different gelators. Certainly, this simple “mixing” strategy requests relatively stable supramolecular gels with tolerant features. Otherwise adding additional porphyrin/phthalocyanine molecules into the systems could destroy the supramolecular gels. And the interactions between mixed porphyrin/phthalocyanine molecules and gelators should be considered. Depending on the solvents included into the supramolecular gels, these mixed porphyrin/phthalocyanine molecules either can be dissolved into the liquid phase or form aggregations within the gels systems. Even though the solubility of porphyrin/phthalocyanine components and complex non-covalent interactions should be considered, the advantage of this simple “mixing” approach is still obvious. Thus, porphyrin and phthalocyanine with different molecular structures can be included into the supramolecular gels.

Only little works were performed on the development of porphyrin or phthalocyanine molecules as the complementary ingredient for the formation of gelators. Considering the difficulty involved in the synthesis processes, sometimes synthesis of porphyrin/phthalocyanine based gelators should be easier than that of developing complementary molecular pairs. What is worth noticing is α-cyclodextrin (α-CD), which help the amphiphilic porphyrin-cored, star-shaped poly (e-caprolactone)-b-poly (ethylene glycol) (SPPCL-b-PEG) copolymer (**17**) form thixotropic and reversible supramolecular hydrogels (Figure 9A; Jin et al., 2015). Moreover, hydrogel nanocomposites systems were also fabricated by using the co-assembly of α-cyclodextrin, PEG-conjugated porphyrin, and multi-walled carbon nanotubes (Figure 7C; Liang et al., 2017b). By connecting α-cyclodextrin with photothermal dyes and further co-assemble with PEG-conjugated porphyrins, photothermally controllable, visible, dual fluorescent thermosensitive hydrogels were developed (Yang et al., 2018a). Except for porphyrin derivatives, cyclodextrin was also applied for the gelation of phthalocyanine systems. For example, multi-photoresponsive supramolecular hydrogels have been developed by mixing poly-β-cyclodextrin polymer, hydrophobically modified dextran, zinc phthalocyanine, and a tailored nitric oxide photodonor (Figure 10A; Fraix et al., 2014).

**Figure 4:**
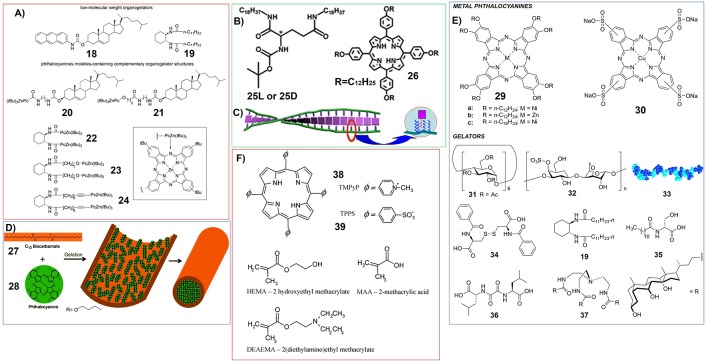
**(A)** Molecular structures of organogelators (18, 19) and zinc phthalocyanine containing complementary organogelators (20-24); Reprinted with permission from Díaz et al. (2008). Copyright 2008 John Wiley and Sons. **(B)** Molecular structures of glutamic acid-based gelators (25) and achiral porphyrin with long alkyl chains (26); **(C)** Schematic illustration showing the formation of induced supramolecular chirality of 26 upon the gelation with 25; Reprinted with permission from Li et al. (2007). Copyright 2007 Royal Society of Chemistry. **(D)** Schematic illustration showing the encapsulation of phthalocyanine (28) in the hollow tubes of biscarbamate (27) gels.; Reprinted with permission from Khan and Sundararajan (2011). Copyright 2011 John Wiley and Sons. **(E)** Structures of metallo-phthalocyanines and gelator molecules. 33 is the schematic structure of a protein which can form hydrogels.; **(F)** Molecular structures of charged porphyrins (38, 39) and monomers used for making polymer hydrogels.

For another type of supramolecular gel based on complementary phthalocyanine, both the phthalocyanine molecules and their complemental molecular systems have gelation substituents. The thermoreversible organogels were prepared by using combination of low-molecular-weight organogelators (**18**, **19**) and zinc phthalocyanine moieties containing complementary organogelator (**20**-**24**). And further *in situ* cross-linking could enhance the strength of the gels (Figure 4A; Díaz et al., 2008).

On the other hand, “mixing” strategy has been used more widely for the fabrication of multicomponent supramolecular gels containing porphyrin or phthalocyanine molecules. Certainly, the careful molecular design for the good gelators is necessary for this strategy. The most representative gelator can be some glutamic acid derivatives, for example N,N'-bis(octadecyl)-L-Boc-glutamic diamide (**25**-**L**) and N,N'-bis(octadecyl)-D-Boc-glutamic diamide (**25**-**D**) (Figure 4B). **25** can form supramolecular gel in nearly all kinds of organic solvents. And the achiral porphyrin with long alkyl chains (**26**) has been included upon mixing without damaging the mechanical strength of the corresponding gels formed in DMSO. Most interestingly, these glutamic acid-based gelators can induce achiral porphyrin form supramolecular chiral assemblies (Li et al., 2007).

Except for glutamic acid-based gels, the hydrogels formed by aromatic short peptide Fluorenylmethoxycarbonyl-Leucine-Leucine-Leucine-OMe (Fmoc-L3-OMe) can also be doped with zinc porphyrin. And enhanced photocurrent generation was investigated from this hydrogel system (Figure 13A) (Feng et al., 2018).

A similar approach was developed by using organogel fabricated by (1R,2R)-trans-1,2-bis(dodecanoylamino)cyclohexane. When octakis(alkyloxy)-substituted Zn(II)-phthalocyanines were incorporated into the gels, unique brush-like nanostructures can be detected (Diaz Diaz et al., 2007). A non-chiral organogelator (**27**) was also found to form gels with hollow fibrous nanostructures, which can encapsulate phthalocyanine molecules (**28**). And phthalocyanine molecules (**28**) form crystal within the gels (Figure 4D; Khan and Sundararajan, 2011). In another sample, hydrogels based on the mixture of sodium deoxycholate and lysine hydrochloride were included with magnesium phthalocyaninate containing crown ether substituents (Goldshleger et al., 2018).

Recently, non-covalent inclusion of different metallo-phthalocyanines into different gel networks have been thoroughly investigated (Figure 4E; Keseberg et al., 2016). The results show that adding metallo-phthalocyanines could change the thermal, morphological, and mechanical properties of supramolecular gels.

Polymer gels always play very important roles for including porphyrin and phthalocyanine molecules. For example, charged porphyrins TMPyP (tetrakis(4-N-methylpyridyl)porphyrin) (**38**) and TPPS (tetrakis(4-sulfonatophenyl)porphyrin) (**39**) have been doped into the polymer hydrogels fabricated by copolymers of HEMA (2-hydroxyethyl methacrylate) with either MAA (methacrylic acid) or DEAEMA (2-(diethylamino)ethyl methacrylate) (Figure 4F). Upon photoirradiation, the porphyrin molecules (**38**, **39**) included in the hydrogels can generate singlet oxygen (Brady et al., 2007).

Doping with different functional molecules is one of the distinctive features of polymer gels. And different polymer gels systems, which can be fabricated by polypyrrole (Wang et al., 2015b), chitosan (Karimi and Khodadadi, 2016; Xia et al., 2017) or cellulose derivatives (Yang et al., 2018b) have been doped with porphyrin and phthalocyanine molecules. And the applications of these functional systems will be discussed in the following of this article.

## Functions of Supramolecular Gels Containing Porphyrin and Phthalocyanine

As a type of characteristic soft matter, porphyrin/phthalocyanine based supramolecular gels have great potential for different applications, from biomedical systems to optoelectronic devices. Even though we cannot address every aspect of these functions in this manuscript, we still want to show some typical new advances of these issues. Since attentions have been focused on the mechanical properties of supramolecular gels, tuning mechanical properties of supramolecular gels containing porphyrin and phthalocyanine molecules will be discussed. Moreover, the concerns on the supramolecular chirality of porphyrin/phthalocyanine based supramolecular gels also will be addressed in this review.

The photophysical, photochemical and electrochemical properties of porphyrin/phthalocyanine based supramolecular gels will be discussed. And the applications of these soft matters could be found in the fields of photodynamic therapy, molecular sensing, luminescence and cell imaging, photocurrents and semiconducting. Moreover, the catalytic properties of gels containing porphyrin and phthalocyanine molecules are also very interesting. These gels-based catalysts have been used for photocatalytic hydrogen production, some organic reactions, and even mimicking enzymes *in vivo*.

### Mechanical Properties of Porphyrin/Phthalocyanine Based Supramolecular Gels

**Figure 5:**
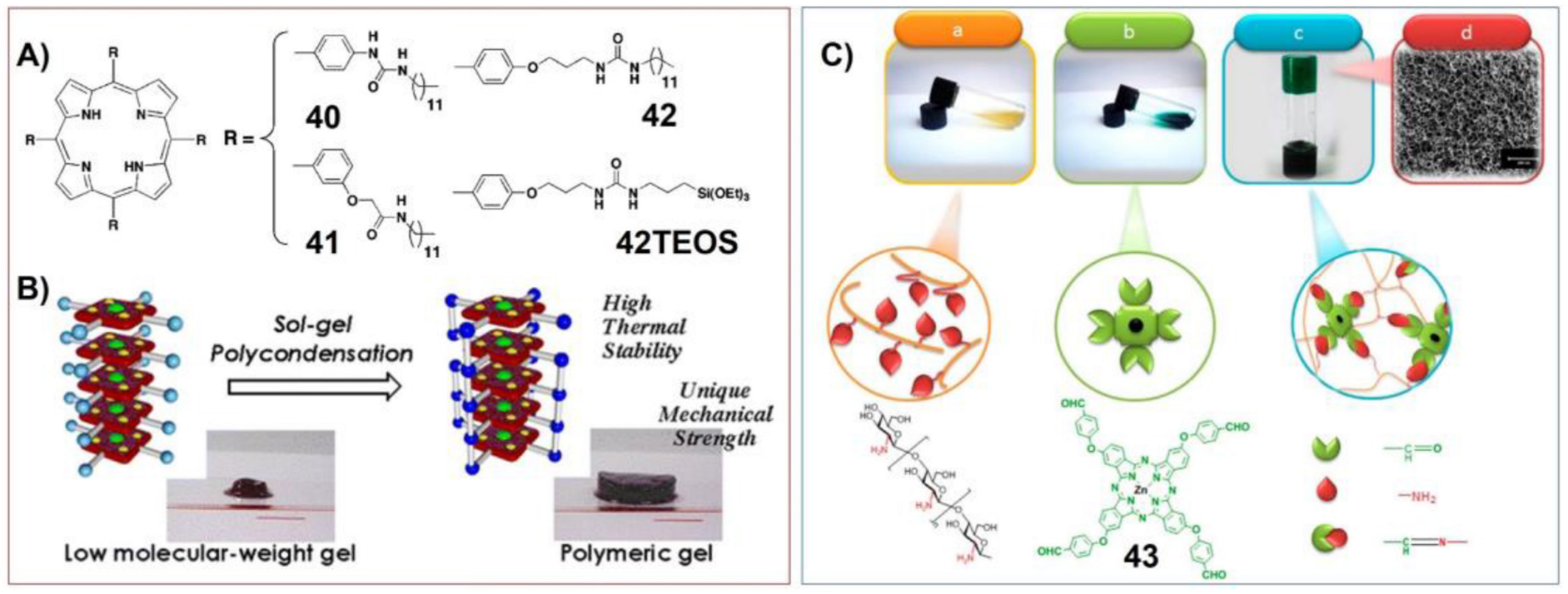
**(A)** Molecular structures of porphyrin gelators containing triethoxysilyl substituents; **(B)** schematic illustration showing the sol-gel polycondensation of the peripheral triethoxysilyl groups of porphyrin-based gelators, which change supramolecular gels into organic/inorganic hybrid materials with unique mechanical strength; Reprinted with permission from Kishida et al. (2005b). Copyright 2005 American Chemical Society. **(C)** Photograph and chemical structure of (a) chitosan and (b) zinc phthalocyanine containing tetra-aldehyde substituents (43) before gelation. (c) Photograph and schematic of a proposed structure of the phthalocyanine/chitosan hydrogel through Schiff-based reaction. (d) Pore size of 3D porous nanostructure phthalocyanine/chitosan hydrogel. Reprinted with permission from Karimi and Khodadadi (2016). Copyright 2016 American Chemical Society.

One of the important characteristics of supramolecular gels is their mechanical properties, which can be the tunable strength, self-healing properties and so on. The mechanical properties of porphyrin/phthalocyanine based supramolecular gels have been investigated by scientists for a long time. The original idea could be developing robust materials containing porphyrin. For example, Shinkai and his co-workers have synthesized the porphyrin gelators containing triethoxysilyl substituents (**42TEOS**) (Figure 5A). The interactions between porphyrin and copper ions could form supramolecular gels with H-aggregated one-dimensional molecular assemblies, which can be further immobilized by sol-gel polycondensation of the peripheral triethoxysilyl groups. Therefore, by using porphyrin-based supramolecular gels as the template, novel functional organic/inorganic hybrid materials with very high thermal stability as well as a unique mechanical strength can be prepared (Figure 5B; Kishida et al., 2005a,b).

Moreover, when phthalocyanine molecules were integrated into the polymer hydrogels, the materials with mechanically robust 3D nanostructure and self-healing properties can be fabricated. Karimi and his co-workers have synthesized zinc phthalocyanine containing tetra-aldehyde substituents (**43**), which can crosslink covalently with chitosan containing amino groups to form dynamic schiff-base linkage (Figure 5C). The corresponding phthalocyanine/chitosan frameworks are hydrogels with 3D porous nanostructure and self-healing ability. Moreover, further including carbon nanotubes (CNTs) could modulate the mechanical properties and electrical conductivity of the hydrogels (Karimi and Khodadadi, 2016).

### Supramolecular Chirality of Porphyrin/Phthalocyanine Based Supramolecular Gels

Chirality is the basic characteristics of nature. And the origin and evolution of life is closely related to the molecular chirality and supramolecular chirality (Michaeli et al., 2016; Pizzarello, 2016). The research on chirality is significant for understanding many of the secrets of nature. Notably, chiral information can be expressed within different scales, from molecules to supramolecular assemblies until macroscopic scales. Constructing assemblies with supramolecular chirality is very useful for producing novel soft matters with different potential applications (Liu et al., 2015). For supramolecular gels containing porphyrin/phthalocyanine, the research works on chirality transfer and amplification are very important.

**Figure 6:**
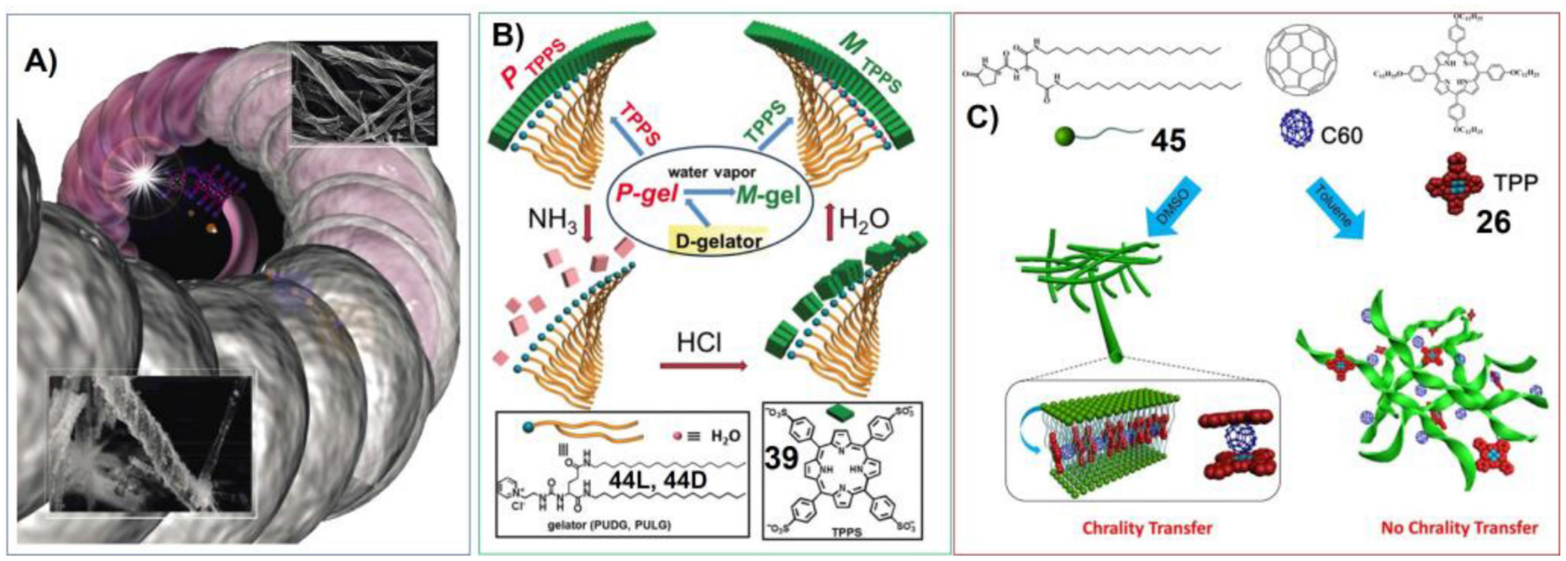
**(A)** Porphyrin-based hydrogels working as template to manufacture helical silica; Reprinted with permission from Kawano et al. (2004). Copyright 2004 John Wiley and Sons. **(B)** Molecular structure of gelator and porphyrin. 44D can form gel with P-chirality, which changes into M-chiral gel upon water vapor. Porphyrin 39 co-assemble with 44D into J aggregates with P helical packing. NH_3_ can destroy porphyrin assemblies, while subsequent treatment with HCl and water vapor can reassemble 39 into M-chirality; Reprinted with permission from Wang et al. (2016). Copyright 2016 Royal Society of Chemistry. **(C)** Molecular structures of the glutamic acid-based gelator containing two chiral centers (45) and achiral C60 and porphyrin (26), and a schematic illustration of the self-assembly of the two achiral molecules with the gelators. Chirality transfer occurred in the DMSO gel but not in the toluene gel. Reprinted with permission from Li et al. (2017b). Copyright 2017 John Wiley and Sons.

These situations are manifested in the transcription of helical assemblies into helical-silica structures. Shinkai et al have synthesized the porphyrin-based gelators containing carbohydrate substituents. And the self-assembly of these sugar-appended porphyrins can form different helical structures depending on the molecular structure and chirality of carbohydrate groups. Upon Sol-Gel polycondensation of tetraethyl orthosilicate (TEOS), the organic helical nanostructures can be transcripted into helical-silica structures, which nicely inherit the organic morphology (Figure 6A; Kawano et al., 2004).

For the chiral-related application of porphyrin/phthalocyanine based supramolecular gels, understanding the chirality transfer within these systems could be significant. Sometimes, when the chiral gelators self-assemble into helical nanostructures, the handedness of their molecular chirality and the corresponding supramolecular chirality could be different. In this context, the optical activity of doped porphyrin assemblies could be interesting. Liu et al. have designed cationic gelators bearing pyridinium and glutamide moieties (**44D** or **44L**). And the co-assembly of these cationic chiral gelators with dianionic tetrakis(4-sulfonatophenyl)porphyrin (**39**) could form J-aggregates. Interestingly, the chiral signs of porphyrin assemblies were found to follow the supramolecular chirality of **44** assemblies instead of the molecular chirality of **44** (Figure 6B; Wang et al., 2016).

The chirality transfer, inversion and amplification within more complex supramolecular gels were further investigated by Liu et al. The glutamic acid-based gelator containing two chiral centers (**45**) was found to form chiral nanotwists and nanotubes in toluene and DMSO, respectively. While the co-assembly of **45** with achiral porphyrin containing long alkyl chains (**26**) and C60 can also form gel in DMSO by keeping the tubular nanostructures. Both porphyrin and C60 were included in confined nanotubes. Chirality transfer occurred from the host chiral gel matrixes to guest achiral porphyrin in DMSO. Remarkably, the addition of C60 to the porphyrin/gelator gel could invert and further amplify the induced chirality of the porphyrin due to the formation of donor-acceptor pairs. However, in the case of multicomponent toluene gel, **26** and C60 only dissolve in the liquid phase. And no chirality transfer was observed in the toluene gel (Figure 6C; Li et al., 2017b).

### Porphyrin/Phthalocyanine Based Hydrogels for Biological Imaging

Porphyrin molecules usually show strong fluorescence, which has great potential for biological imaging. On the other hand, hydrogels have been widely used in the field of biology and medicine. Therefore, when porphyrin molecules were doped into hydrogels, the first application to be developed can be biological imaging.

**Figure 7:**
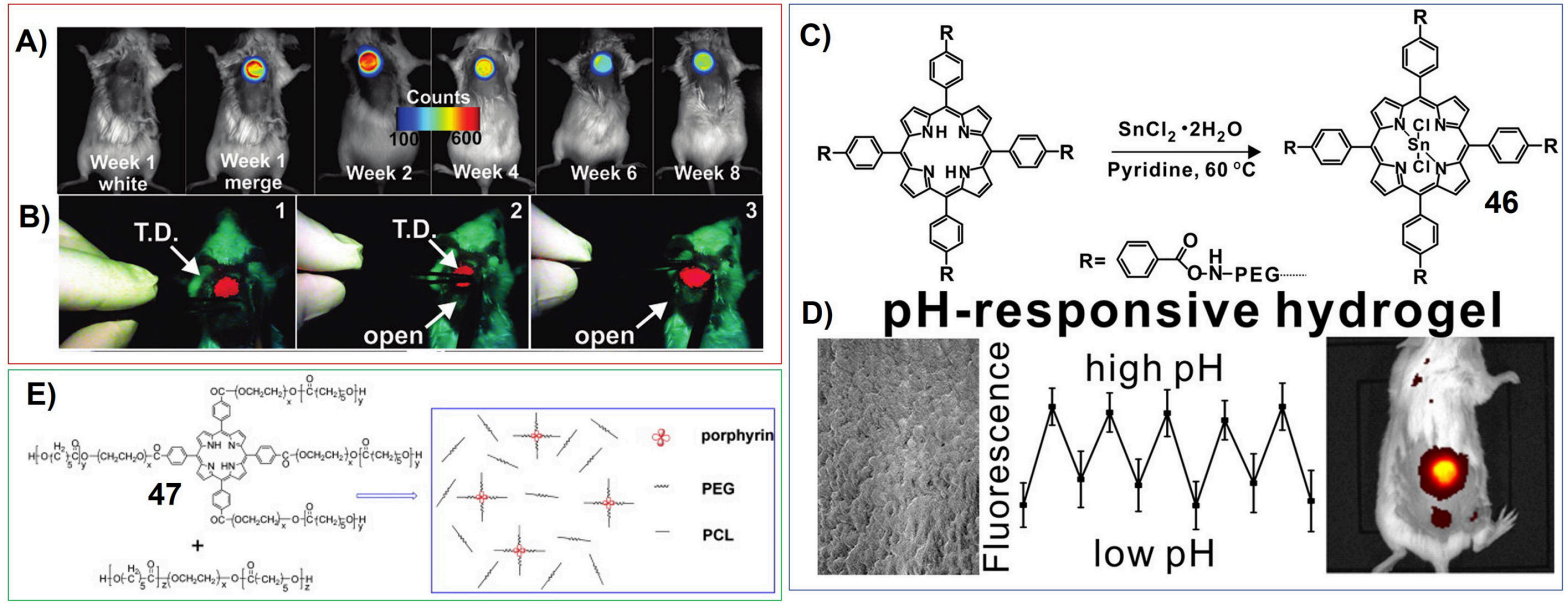
**(A)** Fluorescence images of a mouse with the hydrogel implanted subcutaneously and monitored noninvasively; Reprinted with permission from Lovell et al. (2011). Copyright 2011 American Chemical Society. **(B)** Screen captures from a fluorescence camera used to guide fluorescently the surgical removal of the hydrogel in real time; Reprinted with permission from Lovell et al. (2011). Copyright 2011 American Chemical Society. **(C)** Synthesis of tin-porphyrin-PEG hydrogels; Reprinted with permission from Huang et al. (2017). Copyright 2017 American Chemical Society. **(D)** Implantable tin porphyrin-PEG hydrogels with pH-responsive fluorescence *in vivo*; Reprinted with permission from Huang et al. (2017). Copyright 2017 American Chemical Society. **(E)** Molecular structures of porphyrin conjugated poly(ethylene glycol) (PEG) and ε-caprolactone (47). Reprinted with permission from Lv et al. (2014). Copyright 2014 John Wiley and Sons.

For example, Zheng et al. have developed the hydrogels based on the reaction between PEG diamine and tetracarboxylic acid porphyrins (Lovell et al., 2011; Figure 3F). Within the network of corresponding hydrogels, porphyrin molecules were well separated to prevent fluorescence self-quenching. The near-infrared properties of the fluorescence of porphyrin enabled low background, non-invasive fluorescence monitoring of the implanted hydrogel in a mouse *in vivo*, as well as its image-guided surgical removal in real time (Figures 7A,B).

Moreover, the porphyrin-PEG-diamines polymer can be further chelated with tin ions (**46**) (Figure 7C). Interestingly, the fluorescence emission of Tin porphyrin hydrogel is strongly reversible and pH responsive in the physiological range between pH 6 and pH 8. This pH-sensitive emission was detected via non-invasive transdermal fluorescence imaging *in vivo* following subcutaneous implantation in mice (Figure 7D; Huang et al., 2017).

Lv et al. have synthesized zinc phthalocyanine-PEG-alginate copolymer (**15**). And the hydrogels formed by **15** can be further included with another dye molecule (rhodamine). These dual fluorescent hydrogels can be used for *in vivo* near infrared fluorescence imaging (Liang et al., 2017a). In another work, Lv et al. have synthesized porphyrin conjugated poly (ethylene glycol) (PEG) and ε-caprolactone (**47**) (Figure 7E). And the fluorescent nanogel fabricated by **47** can be used for *in vivo* imaging, which targets tumor tissues in hepatoma tumor-bearing mice (Lv et al., 2014; Dong et al., 2016b).

### Hydrogels Containing Porphyrin/Phthalocyanine for Drug Delivery

**Figure 8:**
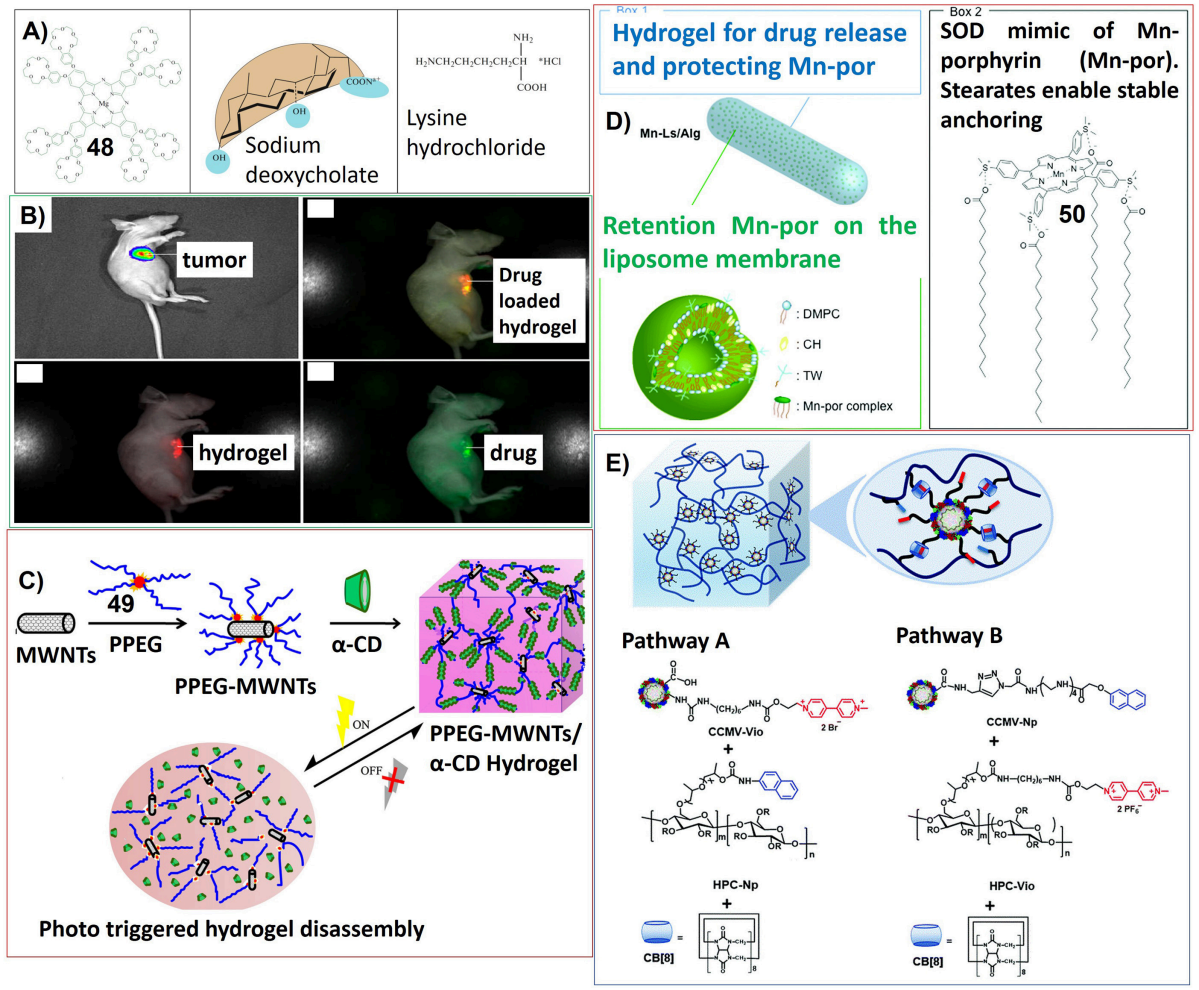
**(A)** Molecular structures of crown-containing phthalocyanines, sodium deoxycholate, and lysine hydrochloride; Reprinted with permission from Goldshleger et al. (2018). Copyright 2018 Pleiades Publishing, Ltd. **(B)** Bioluminescence imaging and multispectral fluorescence imaging for the drug (doxorubicin) loaded hydrogel of mixed fluorescence of the drug and the hydrogel with green and red, representing one of three in each group; Reprinted with permission from Dong et al. (2016a). Copyright 2016 American Chemical Society. **(C)** Schematic of the thermosensitive porphyrin-poly(ethylene glycol) (49)/α-cyclodextrin hydrogel loaded with carbon nanotubes for photo controlled disassembly; Reprinted with permission from Liang et al. (2017b). Copyright 2017 Elsevier Ltd. **(D)** Drug formulation for oral administration of the SOD mimic (50); Reprinted with permission from Aikawa et al. (2015). Copyright 2015 Royal Society of Chemistry. **(E)** Two pathways to prepare compartmentalized supramolecular hydrogels based on virus nanoparticles. Polymeric material HPC and CCMV nanoparticles were cross-linked through a ternary hos-guest interaction, which involved methyl viologen and naphthyl moieties and CB[8]. Reprinted with permission from Yang et al. (2018b). Copyright 2018 Royal Society of Chemistry.

The stimuli-responsive, dynamic properties as well as the biocompatible nature of supramolecular gels render their potential application in the field of drug delivery. For example, the hydrogels based on biodegradable sodium deoxycholate (SDC) and lysine hydrochloride (lys × HCl) can be included with magnesium crown-containing phthalocyanines (**48**), which can be released dependent on thermoreversible of hydrogels (Figure 8A; Goldshleger et al., 2018).

For the gels containing porphyrin and phthalocyanine molecules, sometimes the drug delivery can be related with biological imaging. For example, by using porphyrin-PEG-ε-caprolactone conjugates (**47**) as gelator, the doxorubicin-loaded hydrogel has been developed as dual fluorescent anti-tumor drug delivery system. Using nude mice bearing luciferase expressed hepatic tumor as models, the whole process from the drug delivery to the tumor therapeutic effects were real time visualized simultaneously after administration at interval from 0 to 18 days (Dong et al., 2017). A similar dual fluorescent drug delivery system, which also enables the tracking of a drug delivery process and the degradation of the carrier, was developed by using doxorubicin loaded zinc phthalocyanine (**14**) incorporated hydrogel (Figure 8B; Dong et al., 2016a).

Based on the hydrogel nanocomposites upon co-assembly of α-cyclodextrin, PEG-conjugated porphyrin (**49**) and multi-walled carbon nanotubes, the drug delivery systems with photo response, fluorescence imaging tracking and photothermal remote controlling properties can be achieved (Figure 8C). The controlled disassembly of the hydrogel, which can be efficiently accelerated under laser irradiation, was monitored in real time by *in vivo* fluorescence imaging after subcutaneous injection using mice as models (Liang et al., 2017b). By connecting α-cyclodextrin with photothermal dyes and further co-assemble with PEG-conjugated porphyrins, photothermally controllable, visible, dual fluorescent thermosensitive hydrogels were developed (Yang et al., 2018a). The hydrogel disassembly with drug delivery can be achieved by the photothermal effect of dye molecules. And the dual fluorescence imaging visualization of hydrogels revealed the disassembly process by tracking each component.

For the recently developed drug delivery systems based on porphyrin containing hydrogels, porphyrin molecules can be used as mimic of superoxide dismutase (SOD) inhibiting tumor growth. Yuasa et al. have designed encapsulating liposomal drugs by using an alginate hydrogel as carrier. The liposomal drug was composed of manganese porphyrin (**50**), which has been developed as a mimic of superoxide dismutase (SOD). A cytochrome c assay revealed that the ${\text{O}}_{2}^{•-}$ inhibitory activity of **50** could be maintained even after treatment with simulated gastric and intestinal fluids due to the protection of hydrogels and liposomal aggregations (Figure 8D). The oral administration of the formulated drug significantly inhibited the growth of transplanted tumors in mice (Aikawa et al., 2015).

The most elaborate drug delivery systems based on hydrogels could be the complex of cowpea chlorotic mottle virus (CCMV) particles and guest-modified hydroxylpropyl cellulose (HPC), which were non-covalently crosslinked through the formation of ternary host-guest complexes with cucurbit[8]uril (CB[8]) (Figure 8E). When tetrasulfonated zinc phthalocyanine molecules were loaded in CCMV particles within these hydrogel complex, they showed nice water solubility without undesired aggregation. Moreover, the particles together with phthalocyanine cargo can be released in a controlled way without an initial burst release (Yang et al., 2018b).

### Porphyrin/Phthalocyanine Based Supramolecular Gels for Photodynamic Therapy

When photosensitizers were irradiated with light in the presence of oxygen, free radicals or singlet oxygen could be generated. And photodynamic therapy (PDT) can be achieved by using these active substances for killing tumor cells, bacteria or viruses (Liu et al., 2013; Giuntini et al., 2014; Moylan et al., 2015; Chen et al., 2017; Li et al., 2018a; Zhu et al., 2018). The effects of PDT can be dependent on the photochemical properties of photosensitizers, metabolic characteristic of PDT molecules and accumulation of photosensitizers in the diseased tissue. In this context, both the molecular structure and supramolecular assembly (Li et al., 2018b, 2019a; Zhang et al., 2018) of photosensitizers are important.

Many porphyrins or phthalocyanine molecules have been developed for the photodynamic therapy (PDT) of different illnesses (Singh et al., 2015; Martinez De Pinillos Bayona et al., 2017). On the other hand, porphyrin/phthalocyanine based supramolecular gels for photodynamic therapy not only provides the possibility of controllable photosensitizer delivery, but also help photosensitizer molecules form useful supramolecular nanostructures. The networks of supramolecular gels segment the space within microscopic scale, which brings many unique features for photodynamic therapy. For polymer hydrogels containing porphyrin photosensitizers, the porphyrin molecules can either be included in solid phase or liquid phase. And both the self-assembly of porphyrin and interaction between porphyrin and gelators could affect the corresponding photochemical properties and yield of singlet oxygen (Brady et al., 2007).

**Figure 9:**
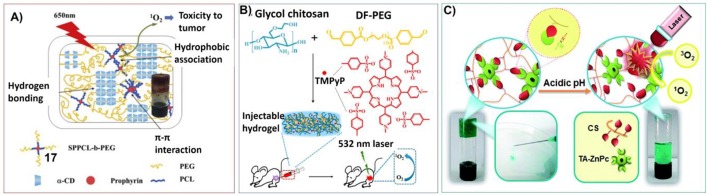
**(A)** Schematic illustration of intermolecular multi-interactions of hydrogels fabricated by 51 and a-cyclodextrin; Reprinted with permission from Jin et al. (2015). Copyright 2015 Elsevier Ltd. **(B)** The synthetic route of the TMPyP(38)-loaded injectable hydrogel and *in vivo* anticancer application; Reprinted with permission from Xia et al. (2017). Copyright 2017 American Chemical Society. **(C)** pH-sensitive and injectable covalently cross-linked hydrogel for *in situ* tetra-aldehyde functionalized zinc phthalocyanine (43) delivery for PDT. Reprinted with permission from Karimi et al. (2016). Copyright 2016 American Chemical Society.

Nevertheless, the applications of hydrogels for the delivery and release of porphyrin for photodynamic therapy are receiving increasing attention. For example, one derivative of α-cyclodextrin, PEG-conjugated porphyrin (**17**) was used for constructing supramolecular hydrogels upon host-guest interaction with a-cyclodextrin (a-CD). The corresponding supramolecular hydrogels have good biodegradable and biocompatible properties, which can be used as carrier for doxorubicin (DOX) delivery systems. Considering the generation of singlet oxygen upon photoirradiation on porphyrin, the hydrogels for both sustained-release drug delivery and photodynamic therapy (PDT) can be achieved (Figure 9A; Jin et al., 2015).

Wu et al. have developed porphyrin included injectable hydrogel by using glycol chitosan and dibenzaldehyde-terminated telechelic poly(ethylene glycol) as well as water soluble porphyrin (TMPyP) (**38**). Comparing to the solution, the hydrogel containing porphyrin shows enhanced fluorescence intensity with much more singlet oxygen generation upon the same laser irradiation. Moreover, much longer tumor retention can be observed due to the low fluidity of the hydrogel (Figure 9B; Xia et al., 2017).

Another injectable hydrogel containing phthalocyanines photosensitizers was developed by Karimi et al. By connecting tetra-aldehyde functionalized zinc phthalocyanine (**43**) with NH_2_ groups on chitosan through a dynamic covalent Schiff-base linkage, pH sensitive photosensitizer delivery hydrogels for localized cancer therapy can be prepared. The hydrogel can release **43** in the acidic environment of tumors directly by evading the circulation system. And the viability of cancer cells incubated with the hydrogel was decreased significantly at acidic pH after laser irradiation (Figure 9C; Karimi et al., 2016).

**Figure 10:**
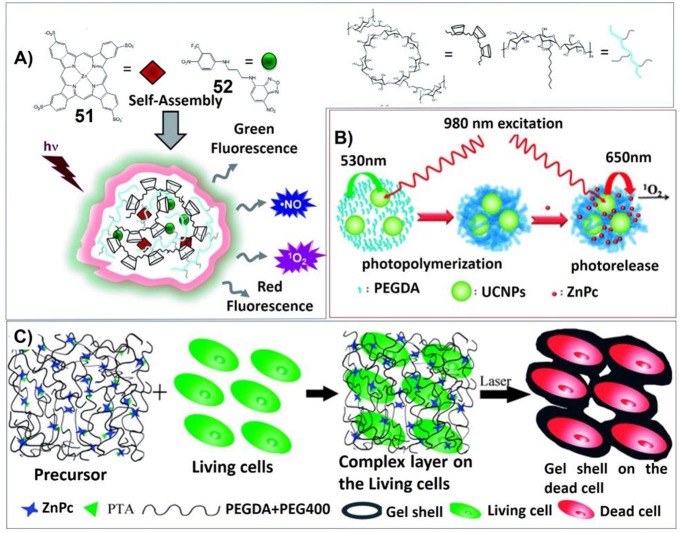
**(A)** The multi-photoresponsive supramolecular hydrogel containing four different components can generate both singlet oxygen and nitric oxide upon visible light irradiation; Reprinted with permission from Fraix et al. (2014). Copyright 2014 Royal Society of Chemistry. **(B)** PEGDA hydrogels containing rare earth-doped nanophosphors (UCNPs) and zinc(II) phthalocyanine. Upon 980 nm irradiation, the multicolor upconversion luminescence of UCNPs can excite photosensitizer zinc(II) phthalocyanine to produce singlet oxygen; Reprinted with permission from Xiao et al. (2013). Copyright 2013 Royal Society of Chemistry. **(C)** Illustrations of the formation process and anti-tumor mechanism of the hydrogel containing poly (ethylene glycol) double acrylates (PEGDA), polyethylene glycol 400 (PEG400), zinc phthalocyanine (ZnPc), and phosphotungstic acid (PTA). Reprinted with permission from Wang et al. (2013). Copyright 2013 Royal Society of Chemistry.

Taking advantages of the inclusiveness of hydrogel systems, multicomponent architectures with more complex functionality can be fabricated for photodynamic therapy. For example, Sortino et al. have developed engineered supramolecular hydrogel containing four different components: poly-b-cyclodextrin polymer, hydrophobically modified dextran, zinc phthalocyanine (**51**) and tailored nitric oxide photodonor (**52**). Upon visible light irradiation, both singlet oxygen and nitric oxide can be generated for photodynamic cancer and bacterial therapies (Figure 10A; Fraix et al., 2014).

In another system, Lin et al. have synthesized poly(ethylene glycol) diacrylate (PEGDA) hydrogels containing rare earth-doped nanophosphors (UCNPs) and zinc(II) phthalocyanine. Upon 980 nm irradiation, the multicolor upconversion luminescence of UCNPs not only can trigger the polymerization of PEGDA, but also can excite photosensitizer zinc(II) phthalocyanine to produce singlet oxygen (Figure 10B; Xiao et al., 2013).

In another approach, the hydrogel containing poly (ethylene glycol) double acrylates (PEGDA), polyethylene glycol 400 (PEG400), zinc phthalocyanine (ZnPc), and phosphotungstic acid (PTA) was prepared. The polymer hydrogel not only prevents diffusion of the photosensitizer, but also maintains a high zinc phthalocyanine concentration around tumor cells for more effective PDT. And phosphotungstic acid also can help the acceleration of singlet oxygen generation (Figure 10C; Wang et al., 2013).

### Porphyrin/Phthalocyanine Based Gels for Molecular Sensing

Some supramolecular assemblies can be used for sensing ions, gas or small organic molecules. For these applications, the interactions between sensors and target molecules could produce some special signals, such as electrical signals or fluorescence (Che et al., 2010; Wong et al., 2010; Saha et al., 2016; Chen et al., 2018). And the molecular sensing can be achieved by analyzing the changes of these signals. Notably, such sensing is dependent on both the molecular structures and the supramolecular properties of the sensors.

**Figure 11:**
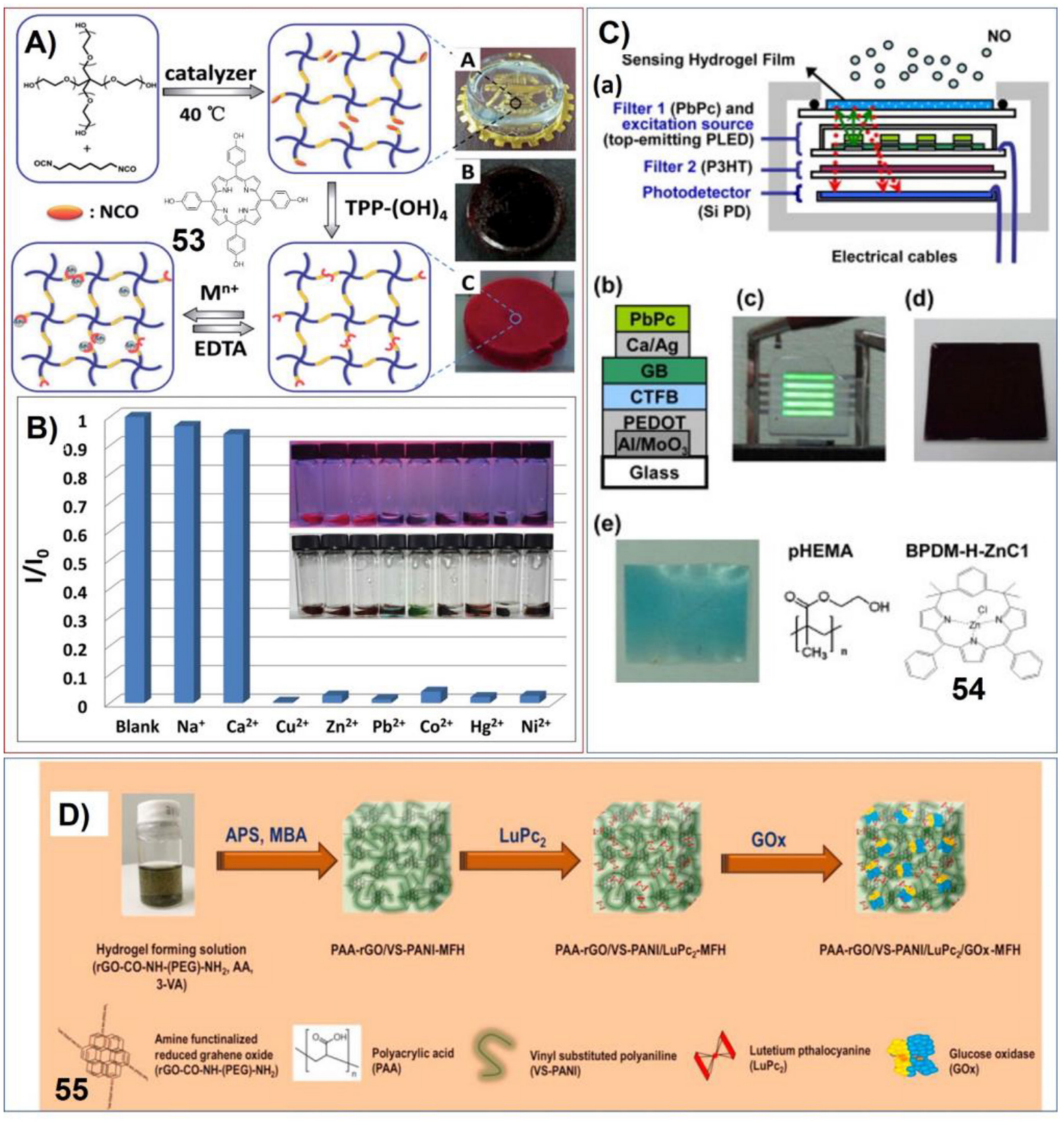
**(A)** Preparation procedure of the PEG-diisocyanate-porphyrin hydrogel; Reprinted with permission from Jia et al. (2015). Copyright 2015 Royal Society of Chemistry. **(B)** Fluorescent intensity of PEG-diisocyanate-porphyrin hydrogels in different metal ion aqueous solutions (0.25 M) at 420 nm. Insert: respective photographs of hydrogels at 365 nm irradiation and visible light (from left to right: blank, Na^+^, Ca^2+^, Cu^2+^, Zn^2+^, Pb^2+^, Co^2+^, Hg^2+^, Ni^2+^); Reprinted with permission from Jia et al. (2015). Copyright 2015 Royal Society of Chemistry. **(C)** Integrated semiconductor optoelectronic devices based on hydrogels for real-time and indicator-free detection of aqueous nitric oxide; Reprinted with permission from Chao et al. (2011). Copyright 2011 Royal Society of Chemistry. **(D)** Schematic representation of the formation of hydrogel (MFH) integrated with reduced grapheme oxide (rGO), vinyl substituted polyaniline (VS-PANI), double-deck lutetium Phthalocyanine (LuPc2), and glucose oxidase (GOx). Reprinted with permission from Al-Sagur et al. (2017). Copyright 2017 Elsevier.

The supramolecular gels containing porphyrin or phthalocyanine have been investigated for molecular sensing. For example, Zheng et al. have fabricated polymeric hydrogels included with porphyrin by connecting 4-arm poly (ethylene glycol) (PEG), 5,10,15,20-tetrakis(4-hydroxyphenyl) porphyrin and hexamethylene diisocyanate (Figure 11A). The corresponding hydrogels show strong fluorescent, which can be further changed upon interaction with heavy metal ions, such as Cu^2+^, Zn^2+^, Pb^2+^, Co^2+^, Hg^2+^, and Ni^2+^. Based on this characteristic, the hydrogel exhibits rapid, significant and visual sensitivity to low concentrations of heavy metal ions (Figure 11B). Moreover, the uptake and release of heavy metal ions can be recycled conveniently (Jia et al., 2015).

Another polymer hydrogel containing porphyrin derivative (**54**) has been prepared into complex devices for real-time and indicator-free detection of aqueous nitric oxide. The polymer hydrogel can be photoluminescence, which is sensing specific to NO. And the changes of photoluminescence can be further transformed into electric signal by the photodetector (Figure 11C; Chao et al., 2011).

Hassan et al. have prepared multifunctional conducting polyacrylic acid (PAA) hydrogel (MFH) integrated with reduced grapheme oxide (rGO), vinyl substituted polyaniline (VS-PANI), double-deck lutetium Phthalocyanine (LuPc2), and glucose oxidase (GOx). This complex system can be used for electrochemical detection of glucose with high sensitivity (15.31 μA mM^−1^ cm^−2^), large concentration range of (2–12 mM) and low detection limit (25 μm). This biosensor based on polymer hydrogel has very fast response time (1 s) and high storage stability (Figure 11D; Al-Sagur et al., 2017).

**Figure 12:**
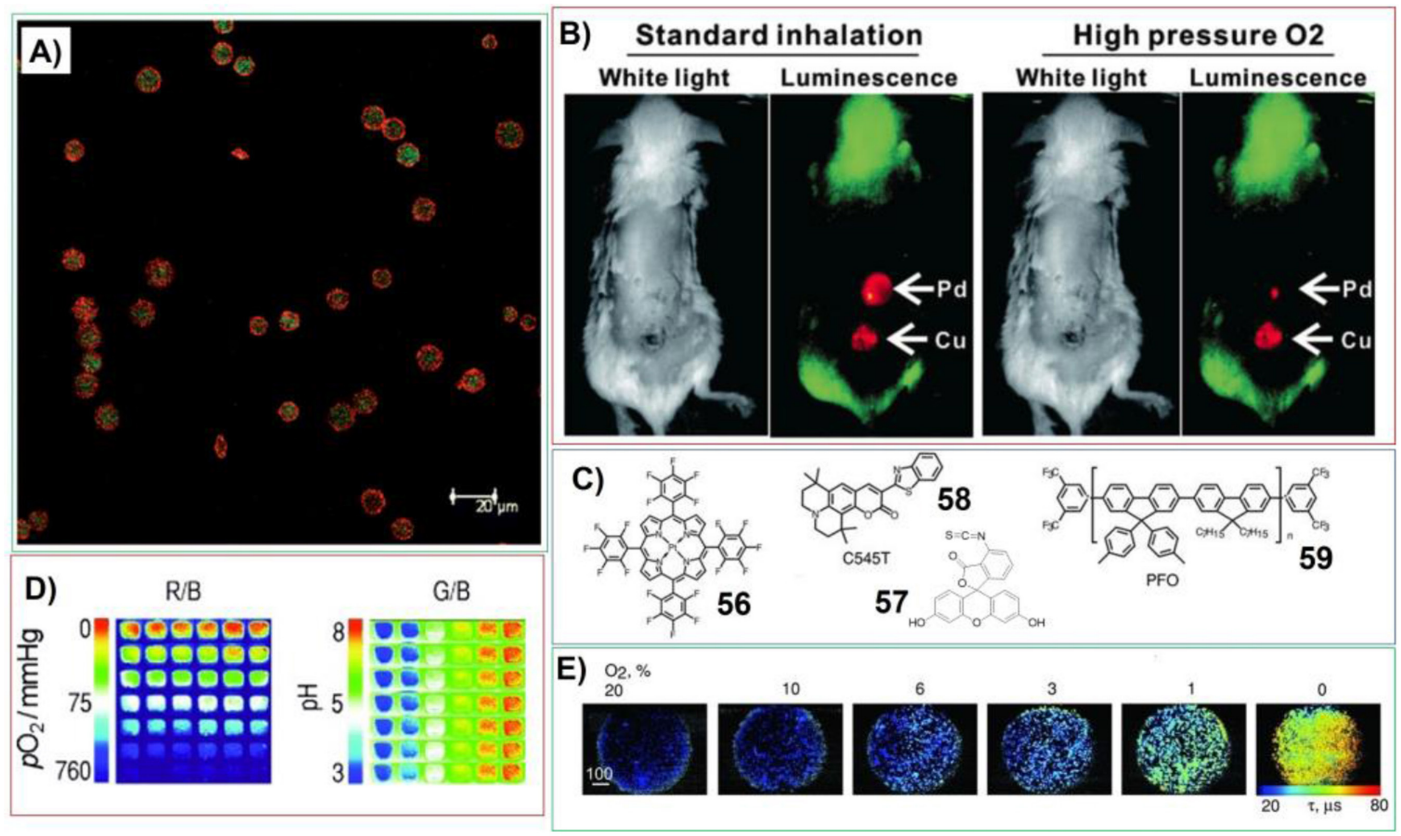
**(A)** Confocal images of microparticle-based ratiometric oxygen sensors (green represents PtP emission; red represents QD emission); Reprinted with permission from Collier et al. (2011). Copyright 2011 Royal Society of Chemistry. **(B)** Transdermal luminescence imaging of dual-implanted Pd-mTCPP and 15% free base-Cu-mTCPP hydrogels. Left images were taken following anesthetization and right ones taken following high-pressure oxygen administration; Reprinted with permission from Huang et al. (2014). Copyright 2014 John Wiley and Sons. **(C)** Molecular structures of components within fluorescent sensor membrane for sensing both pH value and O_2_; **(D)** Sensing process for both O2 and pH. the ratio of red to blue channel pictures (R/B; displayed in pseudo colors) represents the oxygen response and shows the lack of cross-reactivity to pH. The ratio of green to blue channel pictures (G/B; also displayed in pseudo colors) reflects the response to pH and also demonstrates the lack of cross reactivity to oxygen; Reprinted with permission from Meier et al. (2011). Copyright 2011 John Wiley and Sons. **(E)** Widefield fluorescence/phosphorescence lifetime imaging microscopy images of MEF cells stained with MM2 measured at different levels of atmospheric oxygen. Scale bar unit is in μ m. Reprinted with permission from Kondrashina et al. (2012). Copyright 2012 John Wiley and Sons.

Different polymer gels systems containing porphyrins have been developed for gas sensing. For example, McShane et al. have prepared ratiometric oxygen sensor by incorporating two luminophores into polymer hydrogels. In this case, near-infrared emitting quantum dots can be employed as reference luminophores; while long-lifetime platinum (II) porphyrin phosphor was used as oxygen indicator (Figure 12A). This sensing system possess excellent sensitivity (K_SV_ = 0.00826 μM^−1^) at oxygen concentrations below 300 mM and is resistant to photobleaching (Collier et al., 2011). The hydrogel containing porphyrins for *in vivo* oxygen sensing has been developed by Lovell et al (Huang et al., 2014). The reaction between PEG diamine and Pd substituted meso-tetrakis(4-carboxyphenyl) porphine can generate hydrogels with extreme porphyrin density (≈5 × 10^−3^ M) and very weak molecular aggregation. This hydrogel exhibits oxygen-responsive phosphorescence (Figure 12B) and can be stably implanted subcutaneously in mice for weeks without degradation, bleaching, or host rejection. When the matrix containing lots of identical dots included with different luminescent molecules, the fluorescent sensor membrane can be fabricated for sensing both pH value and O_2_ at different emission wavelengths. Wolfbeis et al. have prepared the sensing chips by including platinum(II)-5,10,15,20-tetrakis-(2,3,4,5,6-pentafluorophenyl) porphyrin (**56**), fluorescein isothiocyanate (**57**) (Figure 12C), and the reference fluorophore diphenylanthracene into each dot (Meier et al., 2011). In this case, oxygen can be detected by Pt-porphyrin, while fluorescein isothiocyanate was used to sense pH. The dual sensing can be achieved via RGB (red-green-blue) method, wherein the fluorescence intensities can be imaged with a digital camera (Figure 12D). Nevertheless, sensing cellular oxygen is attracting increased interests recently. By using porphyrin-based hydrogels, more versatile, flexible, and simple oxygen sensors can be fabricated. Papkovsky et al. have developed cell-penetrating phosphorescent nanosensor MM2 by including porphyrin (**56**), fluorescent antennae dyes (**58**) and Förster resonance energy transfer (FRET) donor poly(9,9-dioctylfluorene) (**59**) into cationic hydrogel (Kondrashina et al., 2012). This system provides efficient delivery into the cell and subsequent sensing and high-resolution imaging of cellular oxygen in different detection modalities, including ratiometric intensity and phosphorescence lifetime (Figure 12E).

### Porphyrin/Phthalocyanine Based Gels for Catalyzing

Metal porphyrins or phthalocyanines are good catalysts for different reactions. Many related systems, such as P450, have been thoroughly investigated (Feiters et al., 2000). On the other hand, catalyzing based on gels systems belong to supramolecular catalysis (Feng et al., 2017). When molecules with catalytic properties were self-assembled into ordered nanostructures, their catalytic active centers can also form ordering aggregations. Moreover, the confinement effect derives from self-assembled nanostructures could be very useful for improving the yields and stereoselectivity of chemical reactions. Importantly, the catalytic properties of supramolecular assemblies can be modulated upon changing the solvents and molecular building blocks. In principle, the gels containing porphyrin or phthalocyanine components can be used for catalyzing. And the nature of gel systems should give the advantages for different applications. Although there is still small amount of corresponding results can be found in the literature, the ongoing works already cover different fields. The gels systems containing porphyrin and phthalocyanine have been proved to be able to catalyze some special organic reactions. The photocatalyzing for degradation of contaminants and hydrogen production was also investigated. Moreover, the *in vivo* catalyzing strongly highlights the advantages of the gels.

**Figure 13:**
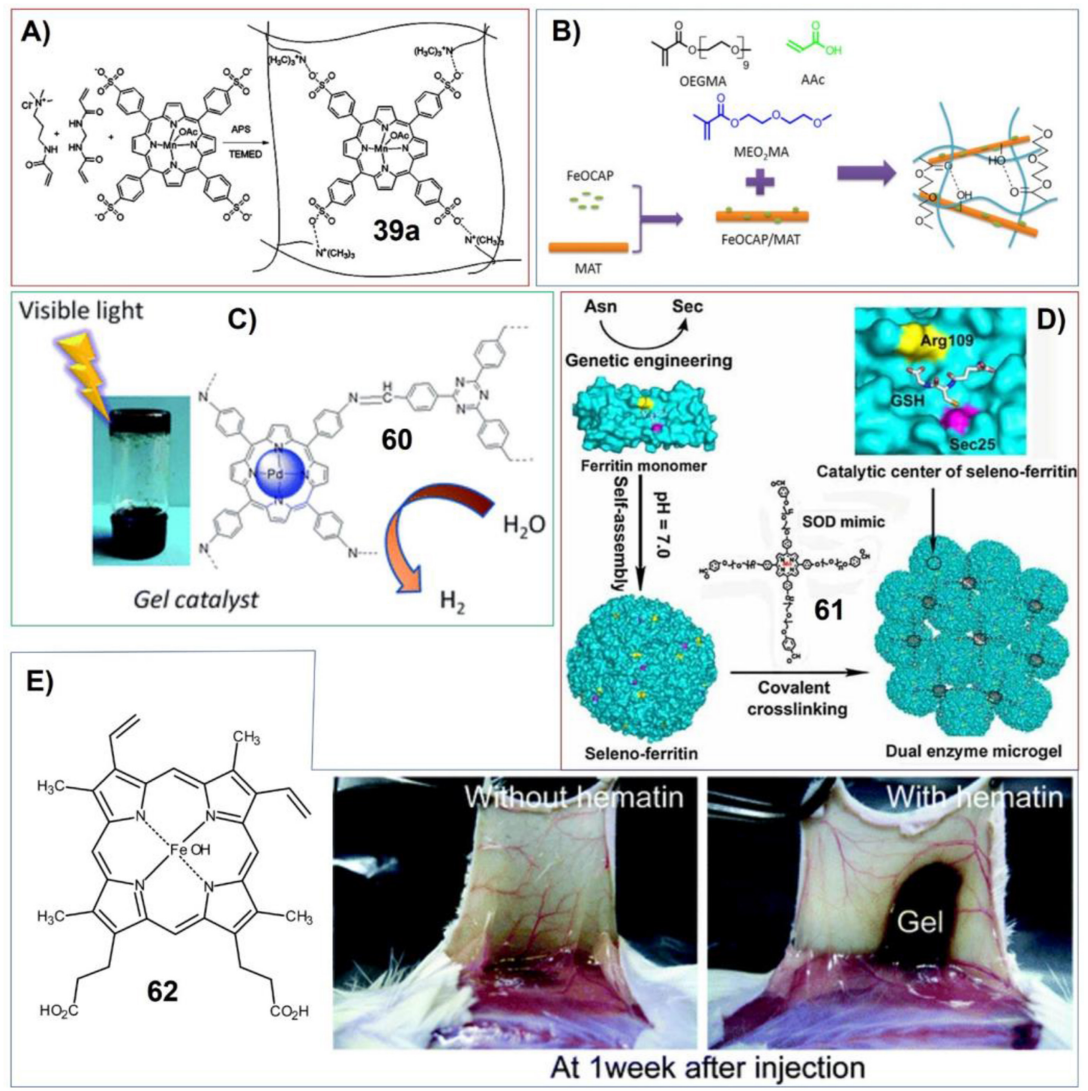
**(A)** Catalyst system comprised of anionic [Mn(tetrakis(4-sulfonatophenyl)porphyrin)(OAc)] ([Mn(TPPS)(OAc)]) embedded within cationic cross-linked polymeric ionic liquid (poly[(3-acrylamidopropyl)trimethylammonium chloride]); Reprinted with permission from Yazdely et al. (2018). Copyright 2018 John Wiley and Sons. **(B)** Poly(ethylene glycol)-based nanocomposite hydrogels containing Fe-octacarboxylic acid phthalocyanine (FeOCAP)/magnetic attapulgite; Reprinted with permission from Yuan and Chen (2017). Copyright 2017 John Wiley and Sons. **(C)** Porphyrin-based imine gels for enhanced visible-light photocatalytic hydrogen production; Reprinted with permission from Liao et al. (2018). Copyright 2018 Royal Society of Chemistry. **(D)** Dual enzyme gel with high antioxidant ability based on engineered seleno-ferritin and artificial superoxide dismutase; Reprinted with permission from Gao et al. (2013). Copyright 2013 John Wiley and Sons. **(E)** Molecular structure of hematin (62), and subcutaneous *in situ* gelation catalyzed by hematin (right). Reprinted with permission from Sakai et al. (2010). Copyright 2010 American Chemical Society.

For catalyzing aerobic oxidation of olefins, Ghorbanloo et al. have developed the hydrogels based on cationic cross-linked polymeric ionic liquid (poly[(3-acrylamidopropyl)trimethylammonium chloride]) embedded with anionic [Mn(tetrakis(4-sulfonatophenyl)porphyrin)(OAc)] (**39a**) (Figure 13A). The activity for catalyzing aerobic oxidation of olefins was found to be dependent on substituent effect of reactants, temperature and the amount of catalysts. And this catalytic hydrogel can be easily recovered from the reaction medium and could be reused for another seven runs without significant loss of activity (Yazdely et al., 2018).

Chen et al. have developed poly(ethylene glycol)-based nanocomposite hydrogels containing Fe-octacarboxylic acid phthalocyanine (FeOCAP)/magnetic attapulgite (Figure 13B). This system has excellent photocatalytic activity for rhodamine B photodegradation. And the hydrogels could be reused more than five times without losing any photodegradation ability (Yuan and Chen, 2017).

An excellent gel system showing enhanced visible light photocatalytic hydrogen production has been developed by Zhang et al. These porphyrin-based imine gels offer chemical variety and hierarchically porous structures. And the properties of these catalysts can be easily medicated by changing porphyrin metal centers. The Pd gel enables efficient photocatalytic H_2_ evolution via photoinjection of electrons from the light-harvesting gel network into the Pt nanoparticles. Upon visible light irradiation, the Pd-porphyrin gels doped with Pt nanoparticles show very high efficiency for hydrogen production in aqueous sodium ascorbate solution (1.0744 × 10^5^ μmol g^−1^ for 120 h totally) without significant degradation during four runs (Figure 13C; Liao et al., 2018).

The catalytic properties of porphyrin-based gels have been utilized for tissue engineering depending on enzyme mimicking. Sakai et al. found that iron-containing porphyrin hematin can act as alternative catalyst to horseradish peroxidase (HRP) for *in situ* gelation of polymers with phenolic hydroxyl (Ph) moieties *in vivo* (Figure 13E). The gelatin derivative with phenolic hydroxyl moieties can form gel in the presence of both hematin and H_2_O_2_ (Sakai et al., 2010).

By making gels containing porphyrin molecules, more complex catalytic systems with delicate nanostructures and functions can be achieved. Liu et al. have fabricated antioxidant microgel with both glutathione peroxidase (GPx) and superoxide dismutase (SOD) activities. In this case, the main catalytic components of glutathione peroxidase were put onto the surface of ferritin. The resulting seleno-ferritin (Se-Fn) monomers can self-assemble into nanocomposites that exhibit remarkable GPx activity due to the well-organized multi-GPx catalytic centers. On the other hand, since some metal porphyrin molecules are good superoxide dismutase mimic, the gel system mimicking synergistic dual enzyme can be prepared by crosslinking seleno-ferritin nanocomposites with porphyrin (Figure 13D; Gao et al., 2013).

### Photoelectric Device Based on Porphyrin or Phthalocyanine Gels

The supramolecular gels containing porphyrin or phthalocyanine building blocks could provide the porphyrin assemblies or phthalocyanine aggregates with nice nanostructures and tailored molecular packing modes. For example, the tetrapyrrole macrocycle based π-conjugated systems can form either J-aggregation or H-aggregation. Different molecular packing modes could render the assemblies with varied spectral characteristics, special energy transfer or electron transfer properties. Therefore, some unusual photophysical, photochemical or electrochemical properties of these assemblies can be expected.

**Figure 14:**
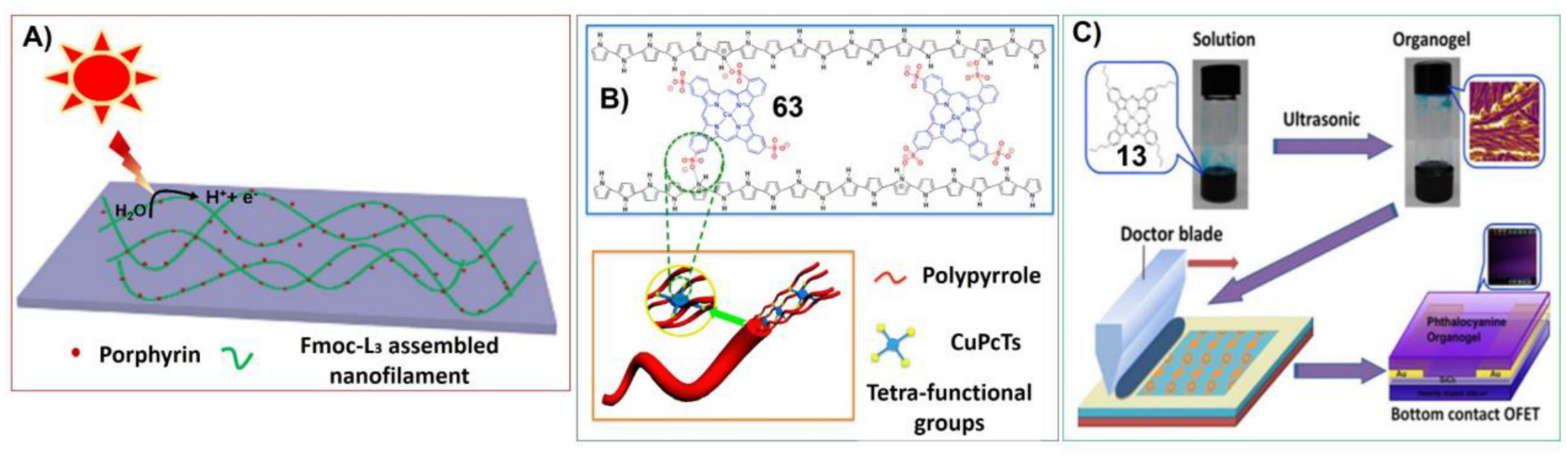
**(A)** Self-assembled peptide hydrogel with porphyrin as a dopant for enhanced photocurrent generation; Reprinted with permission from Feng et al. (2018). Copyright 2018 Elsevier. **(B)** Dopant-enabled supramolecular approach for controlled synthesis of nanostructured conductive polymer hydrogels; Reprinted with permission from Wang et al. (2015b). Copyright 2015 American Chemical Society. **(C)** Ultrasound-induced formation of phthalocyanine organogel for field-effect transistor applications. Reprinted with permission from Xu et al. (2016). Copyright 2016 American Chemical Society.

For example, Bai et al. have fabricated the hydrogels upon the co-assembly of 5,10,15,20-Tetraphenyl-21H,23H-porphine zinc (TPP-Zn) and aromatic short peptide Fluorenylmethoxycarbonyl-Leucine-Leucine-Leucine-OMe (Fmoc-L_3_-OMe). These assemblies show great light response properties. In this case, the long-range order assembly of aromatic peptide can act as photoelectron acceptor and conductor to promote the production of photoelectrons by the light antenna porphyrin molecules. And porphyrin can effectively reduce the resistance, facilitating the transfer of photoelectrons (Figure 14A; Feng et al., 2018).

On the other hand, when porphyrin or phthalocyanine molecules were doped into the supramolecular systems, they can also change the aggregation properties of other molecular building blocks. Polypyrrole is a type of conducting polymer. While doping copper phthalocyanine-3,4′,4″,4‴-tetrasulfonic acid tetrasodium salt (**63**) could cross-link polypyrrole to form hydrogels with three-dimensional network. Phthalocyanine favors the formation of polypyrrole nanofibers. And the enhanced interchain charge transport of polypyrrole/**63** hydrogels resulted in greatly enhanced conductivity and pseudocapacitance compared with pristine polypyrrole (Figure 14B; Wang et al., 2015b).

The gels containing metal phthalocyanines not only can be good electric conductor, but also can be nice semiconductor. Xu et al. synthesized tetra-n-butyl peripheral substituted copper(II) phthalocyanine, which can form supramolecular gels upon ultrasonic irradiation. The corresponding gels can be further fabricated into field-effect transistor (Figure 14C). Due to the stronger π-π interactions within the assemblies, organogel exhibits significant increase in charge carrier mobility in comparison with other solution process techniques (Xu et al., 2016).

## Conclusion and Outlook

Fabrication of supramolecular gels containing porphyrins or phthalocyanines molecules represents the efforts to build novel functional soft matters. In this case, functional molecular building blocks could organize into hierarchical nanostructures in a controlled manner. Many interesting features and subsequently novel applications of these materials could be developed depending on ordered nanostructures. Notably, the dynamic and stimuli-responsive nature of supramolecular gels has greatly expanded the application of included π-conjugated systems.

In principle, gels especially hydrogels can be considered as matter of life, due to many similarities between their nature and that of the organisms (Li et al., 2017a). However, making the supramolecular assemblies as complex as the nanostructures of some cell organelles is still very difficult. It is necessary to further understand the complex non-covalent interactions within various materials. Although there are still big challenges to both controlling the nanostructures and improving performance of functional soft matters, the development of gels systems has provided unlimited expectation and possibility (Weiss, 2014). And the fabrication of different gels systems should be more dependent on the principles and methods of biomimetic. Nevertheless, considering the infinite constructing possibilities of supramolecular gels, functional gels containing the hierarchical assembly of porphyrins or phthalocyanines will be further developed in the field of chemistry, nanoscience, and biological applications.

## Author Contributions

All authors listed have made a substantial, direct and intellectual contribution to the work, and approved it for publication.

### Conflict of Interest Statement

The authors declare that the research was conducted in the absence of any commercial or financial relationships that could be construed as a potential conflict of interest.
